# Development of a compensation-aware virtual rehabilitation system for upper extremity rehabilitation in community-dwelling older adults with stroke

**DOI:** 10.1186/s12984-023-01183-y

**Published:** 2023-05-01

**Authors:** Zhiqiang Luo, Audrey Ei-Ping Lim, Ponraj Durairaj, Kim Kiow Tan, Verawaty Verawaty

**Affiliations:** 1 Foshan University, #18 Jiang-wan-yi-lu, Foshan, 528225 Guangdong People’s Republic of China; 2 Singapore Institute of Technology, 10 Dover Drive, Singapore, 138683 Singapore; 3 Nanyang Polytechnic, 180 Ang Mo Kio Ave 8, Singapore, 569830 Singapore

**Keywords:** Virtual rehabilitation, Compensation, Upper extremity, Stroke, Tele-rehabilitation

## Abstract

**Background:**

Compensatory movements are commonly observed in older adults with stroke during upper extremity (UE) motor rehabilitation, which could limit their motor recovery.

**Aim:**

This study aims to develop a compensation-aware virtual rehabilitation system (VRS) that can detect compensatory movements and improve the outcome of UE rehabilitation in community-dwelling older adults with stroke.

**Methods:**

The VRS development includes three main components: (1) the use of thresholds for determining compensatory movements, (2) the algorithm for processing the kinematic data stream from Kinect to detect compensation in real-time, and (3) the audio-visual feedback to assist older adults with stroke to be aware of the compensation. Two studies were conducted following the VRS development, where Study 1 identified the value of thresholds for determining compensatory movements in two planar motor exercises, and Study 2 provided preliminary validation for the developed VRS by comparing two groups undergoing VR training or conventional training (CT) in a community rehabilitation center.

**Results:**

The VRS could effectively detect all determined compensatory movements and timely trigger feedback in response to the detected compensatory movements. The VR participants showed significant improvements in Fugl-Meyer Assessment-Upper Extremity (FMA-UE, p = 0.045) and Wolf Motor Function Test (WMFT, p = 0.009). However, the VR and CT groups had no significant differences in outcome measures.

**Conclusion:**

The VRS demonstrates the ability to detect compensation and the potential of assisting older adults with stroke to improve motor functions. Suggestions are given for further improvements of the VRS to support the older adult with stroke to reduce compensation.

**Supplementary Information:**

The online version contains supplementary material available at 10.1186/s12984-023-01183-y.

## Background

Stroke has been a global healthcare challenge since it is one of the main causes of acquired adult disability in most countries. Most individuals (especially older adults) with stroke are left with perpetual impairments [1], where a significant proportion of them are left with impaired upper extremity (UE) motor impairment [2, 3]. Functional recovery of UE is usually poor, and as many as 50% of stroke patients still have impairments at six months post-stroke [4]. Continual rehabilitation in the community, such as at the elderly-care center or even at home, could support older adults with stroke to re-establish UE functions [5]. However, the short supply of workforce resources in the community limits the therapist-patient interaction time [6] to monitor and motivate them to take a motor intervention.

Virtual rehabilitation systems (VRS), including those commercial gaming systems (such as Wii and Kinect-based video games), are increasing in popularity given their benefits for stroke rehabilitation. These VRS could deliver the individualized, embodied, and immersive training at the required levels of frequency and intensity and increase older adults' engagement and adherence to motor rehabilitation [7]. Especially in the current Covid-19 pandemic, the VRS deployed in the community or at home could support older adults with stroke to take rehabilitation regimens safely and conveniently.

The deployment of VRS in the community is still at its early stage as it faces a few practical challenges and barriers. One challenge with most existing VRS is missing the function of monitoring older adults' compensatory movements in real-time to ensure the quality of movement in UE motor practice. Older adults with stroke gradually adopt various compensatory movements in performing UE functional tasks [8–10]. The compensatory movement is the new movement pattern to accommodate altered constraints to accomplish UE motor tasks. The adoption of compensatory movement could be caused by UE impairments [11, 12] and muscle weakness [13], which could ultimately limit motor recovery [10, 14] and result in secondary impairments such as joint pain and reduced range of motion [15]. For example, when stroke individuals performed the reaching task, Cirstea and Levin [10] found a significant correlation between increased trunk involvement and reduced elbow extension and shoulder flexion. Thus, monitoring and reducing stroke survivors' compensation during motor practice is important in the community context with a shortage of close therapist supervision.

Recent studies have started addressing the compensation in the design of VR-based rehabilitation through applying various motion tracking technology [16–18] or pressure sensors [19, 20] to detect compensatory movements when stroke survivors perform the UE reaching tasks. For example, Foreman and Engsberg [17] utilized one commercial Kinect device in their VRShape system to measure and shape the trunk compensatory movement during repetitive upper limb practices of a reaching task. Besides the trunk compensation, the affected limb could compensate for the lower arm motion in elevating the upper limb [21]. Cai's studies [19, 20] used a pressure mattress to detect the shoulder elevation besides trunk compensations and proposed a simple method to help stroke survivors reduce the detected compensation.

This study aims to develop a compensation-aware VRS for UE rehabilitation in community-dwelling older adults with stroke. Specifically, the VRS should be able to detect both trunk and UE compensations observed in shoulder flexion and shoulder horizontal abduction and improve the outcome of the UE rehabilitation.

The study focuses on two planar motor exercises, shoulder flexion and shoulder horizontal abduction, which are different from the motor tasks examined in previous research. A recent study [22] suggests that therapeutic games should not target practicing activities of daily life (ADLs) but instead practice the missing component. Moreover, research has shown that the planar motor exercise could improve motor ability with less aggravation of shoulder pain and spasticity and provoke less maladaptive compensatory movements [23].

The contributions of this study are twofold. First, it determines the thresholds of compensatory movements observed in the above two planar motor exercises. To our best knowledge, there is no prior study on quantifying the compensatory movements of the two planar motion exercises. Second, it proposes the algorithm to detect compensatory movements and design the audio-visual feedback to assist older adults with stroke to be aware of the compensation.

## Methods

We firstly describe the hardware and software of the developed VRS. Secondly, we introduce Study 1 to quantitatively assess the trunk and UE compensatory movements. Lastly, we introduce Study 2 to provide preliminary validation for the developed VRS in a local community rehabilitation center. The ethics committee of the researchers' institute in Singapore approved the two studies.

## The virtual rehabilitation system

### Hardware

**Fig. 1:**
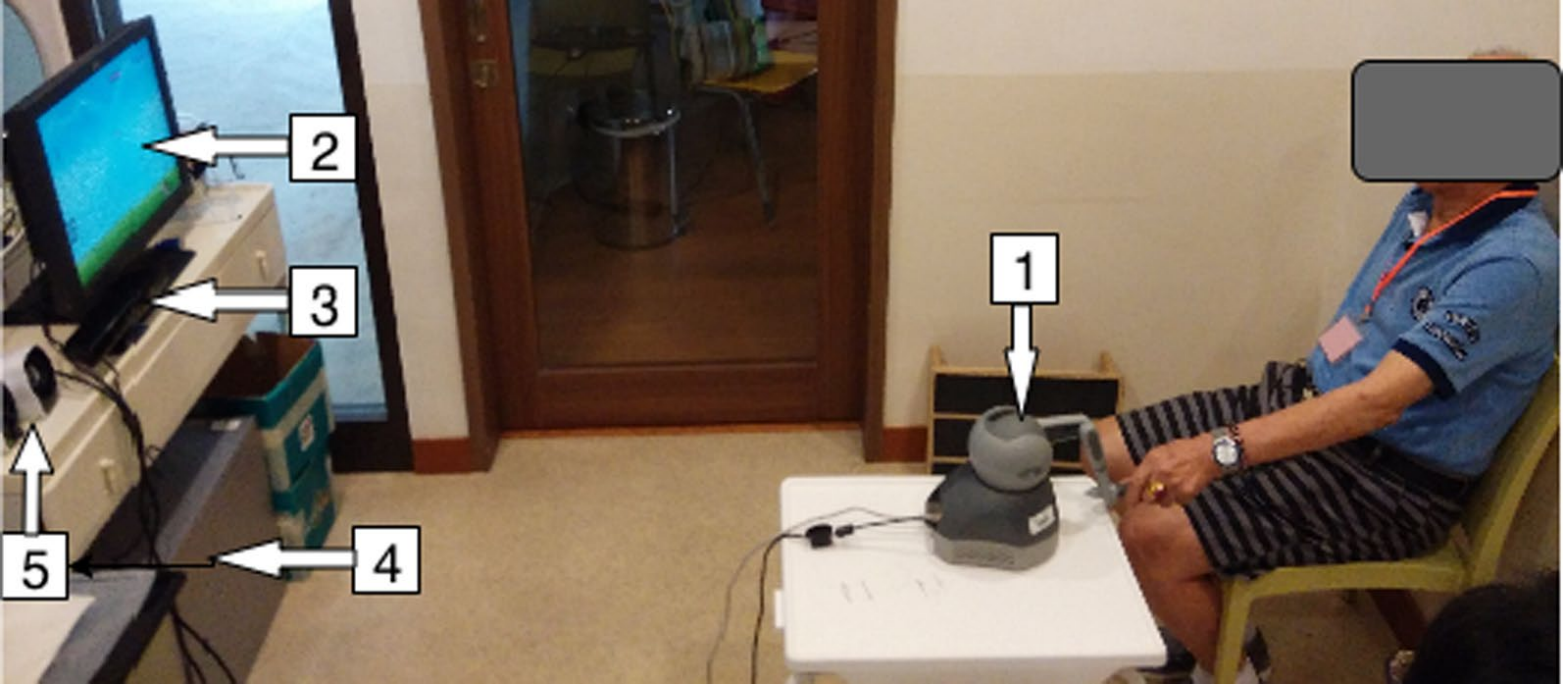
Hardware of VRS set in a community rehabilitation center: 1-haptic device, 2-display, 3-Kinect, 4-PC, and 5-audio box

Figure 1 displays the hardware of a virtual rehabilitation system comprising a Phantom Omni® haptic device (simply called the haptic device hereafter), a Kinect sensor (version 1), a display, an audio box, and a personal computer. The haptic device provided a range of haptic outputs and offered six degrees of freedom allowing the user to manipulate the game objects via operating on the stylus. The Kinect sensor is low-cost, widely available, and easily programmed with the software development kit (SDK). There is empirical evidence that it can accurately assess lateral trunk lean angles during postural control tests [24] and trunk compensatory motion [17] during the reaching task.

### Software

**Fig. 2:**
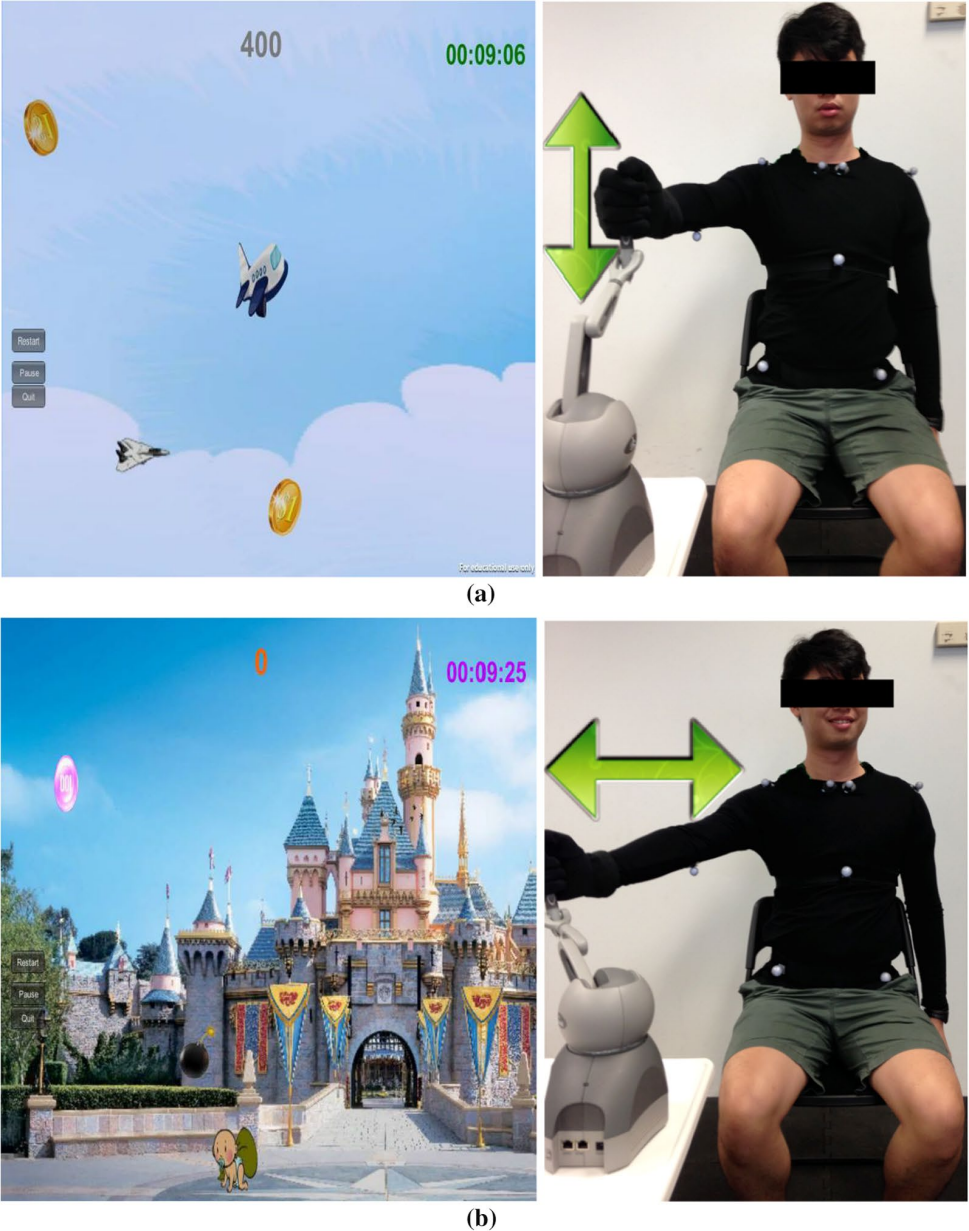
The two rehabilitation games. **a** Shoulder Flexion Game (Game 1, left) and an experimenter holding the stylus of a haptic device to perform shoulder flexion and extension (right) to control the plane in Game 1 to move upward and downward to collect the coins moving horizontally in the game scene and **b** Shoulder Horizontal Abduction Game (Game 2, left) and an experimenter holding the stylus of a haptic device to perform shoulder horizontal abduction (right) to control the character on the ground to collect the coins falling from top to the ground

The software of the VRS mainly comprised a compensation detector, two rehabilitation games including Shoulder Flexion Game (Game 1, shown in Fig. 2) and Shoulder Horizontal Abduction Game (Game 2, shown in Fig. 2), a user profile module, and a visualization module. When playing the Shoulder Flexion Game, a player controls a plane to collect the coins by taking shoulder flexion and extension exercise. When playing the Shoulder Horizontal Abduction Game, a player controls an avatar to collect the coins by taking shoulder horizontal abduction exercise. The two games incorporate the goal-oriented reward scheme since the player's goal is to collect coins to gain the game score. The player’s performance on playing Game 1 could be found in Additional file 1.

### Compensation detector

The compensation detector aims to detect the compensatory movements based on the kinematic data streamed from Kinect and then trigger the corresponding feedback in the gaming scene. The kinematic data collected from Kinect included the orientations and positions of nine joints of the upper body, including the positions of the spine and pelvic, the orientations and position of the chest (called shoulder center in Kinect SDK), right shoulder, left shoulder, the orientations of the right elbow and left elbow, and the positions of right wrist and left wrist. The compensation detector would comprise four steps: calibration, overall detection, precise detection, and feedback trigger.

Step 1-calibration: during calibration, the compensation detector would guide the subject to sit well and hold on to the stylus in the correct and comfortable posture until they confirm starting the motor practice. Then the compensation detector immediately captures the current frame of data stream inputted from Kinect and stores this frame as the calibrated kinematic data.

Step 2-precise detection: precise detection would determine the specific compensatory movement in two ways: (1) comparing the orientation of the selected joint between the real-time kinematic data and the calibrated kinematic data; (2) comparing the distance of the two selected joints between the real-time kinematic data and the calibrated kinematic data. The orientation and distance differences would multiply some constants to be compared with the corresponding thresholds of compensatory movements. Here the thresholds of compensatory movements will be determined in the following Study 1. Lastly, the compensation detector summarizes all comparison results and labels all detected compensatory movements for later processes in Step 3.

1 $${\text{O }} = {\text{ Vector3}}\left( {{\text{ L}}_{{\text{x}}} {-}{\text{T}}_{{\text{x}}} ,{\text{ L}}_{{\text{y}}} {-}{\text{T}}_{{\text{y}}} ,{\text{ L}}_{{\text{z}}} {-}{\text{T}}_{{\text{z}}} } \right)$$

where: O is the orientation, L is the position of spine, T is the position of shoulder center, both positions defined along x, y, z dimensions, respectively

2 $${\text{A }} = {\text{ arcos }}({\text{P}}_{{\text{x}}} \times {\text{ S}}_{{\text{x}}} + {\text{ P}}_{{\text{y}}} \times {\text{ S}}_{{\text{y}}} + {\text{ P}}_{{\text{z}}} \times {\text{ S}}_{{\text{z}}} )$$

where: A is the angle difference, P is the real-time joint orientation, and S is the corresponding joint orientation in calibration, both orientations defined along x, y, z dimensions.

The process to detect the contralateral trunk lateral flexion is illustrated in detail to explain the procedure of precise detection. Firstly, the compensation detector would calculate the orientations from the spine to the shoulder center via Eq. 1. Here the two orientations, O_current_ and O_calibration_, would be calculated, where the positions of the spine and shoulder center would be drawn from the latest frame of a kinematic data stream and the calibrated kinematic data in Step 1, respectively. Secondly, the difference between these two orientations would be calculated according to Eq. 2. Thirdly, the orientation difference would be modified by multiplying one constant **β**. Lastly, the modified orientation difference would be compared with the threshold. If the modified orientation difference result is larger than the threshold, the corresponding compensatory movement (the contralateral trunk lateral flexion) would be labeled in the compensation detector.

Step 3-feedback trigger: this step would trigger the designed audio-visual feedback based on the labeled compensatory movements summarized at Step 2. The compensation detector would end the process until the game scene displays the feedback. The following section introduces the four types of audio-visual feedback.

### Audio-visual feedback

The primary purpose of the audio-visual feedback is to support older adults with stroke to be aware of the compensation during UE rehabilitation. There are four guidelines for designing the audio-visual feedback.

Type 1-alerting player about compensatory movements via text and audio: This type of feedback aims to remind older adults with stroke about compensatory movements straightly. The name of the detected compensatory movement is displayed in the red text with one beep sound in a game scene.

Type 2-increasing the size of the player character: This type of feedback supports older adults with stroke to avoid undesired compensatory movements since the increased size of the player character can reduce the upper limb's range of motion (ROM) to move the stylus to complete the game task.

Type 3-adding additional game objects to induce the player's UE movements: This type of feedback is devised based on operant conditioning [25] that can modify motor behavior through reward and punishment. For example, adding the treasure box into the game scene to reward the correct UE movement, while adding the bomb into the game scene could punish the wrong UE movements.

Type 4-using game scores to differentiate the desired/undesired UE movements: This type of feedback indicates the performance of adapting to the correct UE movement. For example, increasing game scores reward the desired motion, while decreasing game scores punishes the undesired motion.

The last three types of feedback reflect the goal-oriented reward scheme in the VR game design. For example, the increased size of the player character (Type 2) could make it easy to collect coins to achieve high game scores; once the player character collects the treasure box (Type 3), the current game score will be increased.

**Fig. 3:**
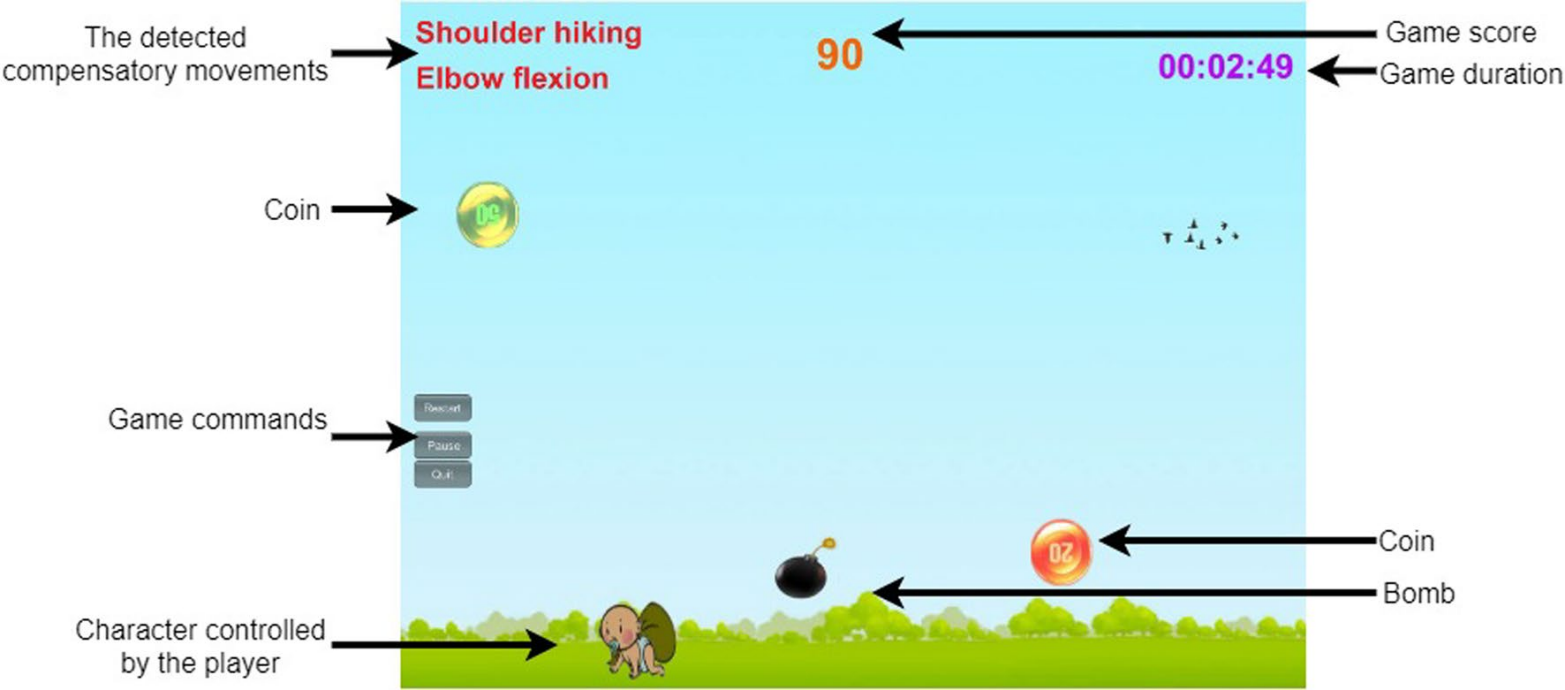
one screenshot of a game scene in Game 2 where the names of all game objects are added

Figure 3 shows one screenshot of Game 2 to explain the above design guidelines. The player would control the character at the bottom to collect the coins falling from the top of the screen. Once the character hits the coin, the game score is increased (Type 4). The names of two compensatory movements are shown in the left-up corner (Type 1). One bomb is shown to prompt the player to pay attention to compensatory movements and avoid the punishment in reducing game scores (Type 3).

## Study 1: quantifying trunk and UE compensations in two planar motion exercises

The purpose of Study 1 was to quantify trunk and UE compensations observed in two planar motion exercises. Specifically, the value of the thresholds for compensatory movements identified in Study 1 could be deployed in the compensation detector.

### Participants

This study employed purposive sampling to recruit participants. Seventeen healthy older adults (7 males, 10 females; age range 58.8 ± 6.33 years) were recruited in public via advertisement. Six hemiparetic stroke participants (3 males, 3 females; age range 67.5 ± 10.4 years) were recruited from a community rehabilitation center. Stroke participants were included if they met the following four conditions: (1) being diagnosed with hemiparesis stroke at least three months previously, (2) being able to remain in a sitting posture (without back/arm rest) for at least 10 min, (3) being able to follow verbal instructions and provide informed consent, (4) being able to voluntarily flex shoulder to 100°, extend elbow to maximum, horizontal abduct and adduct shoulder to 20–25°.

Both healthy and stroke participants were excluded from the study if they were medically unstable or in uncontrolled cardiovascular/ cardiopulmonary conditions, such as uncontrolled hypertension or chronic obstructive pulmonary disease, if they had orthopedic disorder or pain in the upper extremity, or if they had existing joint diseases such as rheumatoid arthritis.

### Experimental setup

The experiment was conducted at one research lab in a local university, where the developed VRS and one commercial marker-based video motion capture (VMC) system, Siliconcoach Pro, would capture participants' motion data at the same time. Previous studies point out the relatively low accuracy of Kinect compared to other VMC systems. The marker-based VMC system is considered the gold standard among motion tracking systems and was used to validate the accuracy of Kinect [16]. Thus the marker-based VMC could quantify trunk and UE compensatory movements more accurately than the Kinect.

The marker-based VMC hardware comprises two cameras (Panasonic 2DR–S71) with a zoom lens adjusted to provide a full view of the participant. Camera One (Frontal View) was placed in line with the sagittal plane, and Camera Two (Sagittal View) was placed perpendicular to the acromion process. A chair with a backrest was placed at the intersection of the two horizontal scaling objects. Participants were seated on the chair with hips and knees flexed to 90^0^ without trunk restraint. Two vertical scaling objects were placed beside the chair in the frontal plane and behind the chair in the sagittal plane. Participants had dressed in a dark shirt with markers attached to identify the joint's location and track motion in the video [26]. The placement of markers was consistent with the International Society of Biomechanics (ISB) Recommendation requirements to achieve high intra-ratter and inter-ratter reliability [27].

The participants were seated in front of the developed VRS. Precisely, the haptic device was placed on a table next to the participants. The table's position was determined at the 45^0^ of shoulder flexion when a subject was able to hold on to the haptic device's stylus while maintaining maximum elbow extension. Specifically, the healthy participants would use the dominant hand to hold on to the stylus, while the stroke participants would use the paretic hand to hold on to the stylus. The distance between the display and the participants were 2 m to ensure that participants could see the scene. The Kinect was placed immediately in front of the display, and its height to the ground was 1 m.

### Experiment procedure

All participants signed an informed consent form before the experiment. Participants were then asked to complete a self-declaration form and a brief physical assessment to gauge the suitability of their participation in the current study. After that, each participant had a one-minute trial of playing the game, and the experimenters would give specific guidance and instruction. Video recording would commence when participants started the formal gameplay. The duration of each game was five minutes. After playing the first game, each participant had a rest for ten minutes and then started playing the second game.

### Data collection and analysis

#### Motion data collected by the VMC system

The motion data collected by the VMC system were processed in a 2D video motion analysis software, Siliconcoach Pro 7.022 application [28] of the VMC system, which has been proved to be a valid and reliable outcome measure [29].

**Fig. 4:**
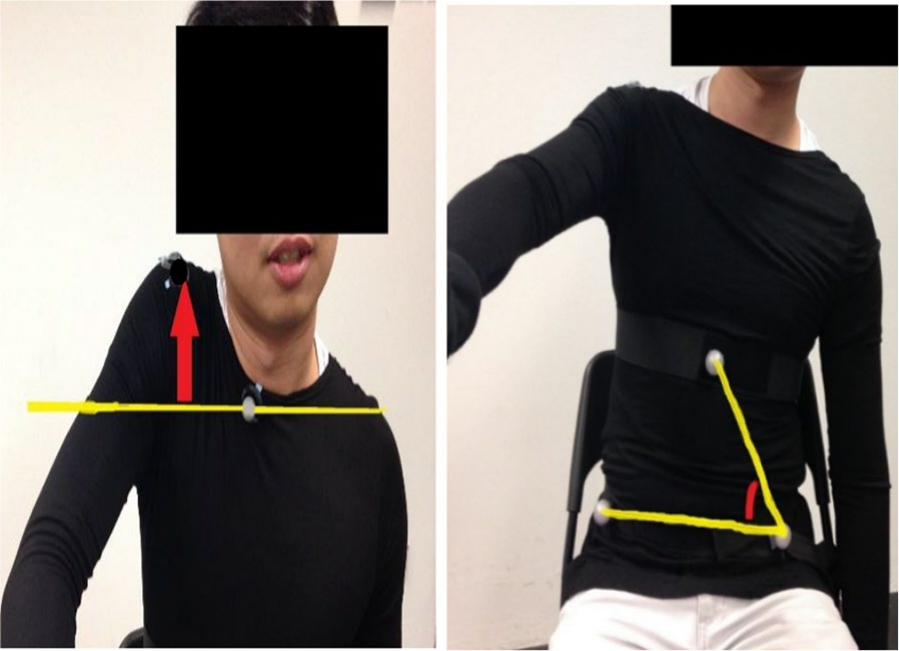
Illustration of measuring the distance of shoulder hiking (left) and the angle of lateral trunk flexion (right) through analyzing the video frame in Siliconcoach Pro software

Figure 4 illustrates the distance of shoulder hiking and the angle of lateral trunk flexion by analyzing one video frame in Siliconcoach Pro 7.022. The distance was measured by drawing one horizontal line across one reference (or stationary) marker, and the distance from the target marker to the horizontal line would then be the length of the vertical line drawn in the application. The angle was measured by drawing a continuous line across three markers in which the reference (or stationary) marker was in the middle, and the angle would then be automatically calculated within the application. The initial distance and angle were measured at the beginning of the shoulder exercise. After finishing each action (e.g., shoulder flexion), the distance and angle were measured again. The difference between two measured distances would be determined as the distance of deviation. The difference between two measure angles would be determined as the angle of deviation.

A group of distances and angles of deviation for trunk, shoulder, and elbow would be collected after one subject finished the five-minute shoulder exercise. Then the maximum and minimum distances and angles of deviation could be found by sorting the group of distances and angles of deviation in descending order. In this study, the maximum/minimum distance and angle of deviation of healthy controls were named maximum/minimum deviation, while the maximum/minimum distance and angle of deviation of stroke participants were named the maximum/minimum "compensation." After all subjects finished two motor exercises, a group of maximum and minimum deviation or compensation of distance and angle would be collected.

The collected deviation and compensation data of distance and angle were analyzed in IBM SPSS Statistics Version 21. The maximum, minimum, median, and interquartile range (IQR) of each joint's compensation or deviation of distance and angle were calculated. Then, the kinematic data of healthy and stroke participants were compared in two ways. First, the maximum compensations in stroke participants were compared to the maximum deviations in healthy control to find the compensation strategies adopted by the stroke participants. Second, the minimum compensations of stroke participants were compared to the maximum deviations in healthy control to determine the thresholds of compensation movements. The Shapiro–Wilk Test of normality revealed a non-normal distribution, and Levene's test of homogeneity of variances of the data showed that the variances were heterogeneous, and thus a non-parametric test, the Mann–Whitney U test, was used to compare the two groups.

## Results

1. Differences between maximum compensation movements in stroke participants and maximum deviations in healthy participants
  Table 1 lists the distance and angle differences between the maximum compensations in stroke participants and the maximum deviations in healthy controls. When playing the shoulder flexion game, the stroke participant’s maximum compensation movements were significantly larger than the healthy control’s maximum deviation in ipsilateral shoulder hiking (z = − 2.27, p = 0.025, d = 0.47), ipsilateral shoulder depression (Z = − 2.89, p = 0.003, d = 0.60), contralateral shoulder depression (z = − 3.93, p = 0.000, d = 0.82), contralateral trunk lateral flexion (z = − 2.80, p = 0.008, d = 0.58), and ipsilateral elbow flexion (z = − 2.21, p = 0.025, d = 0.46). It should be noted that the contralateral shoulder depression (Healthy = 0.000 m, Stroke = 0.015 m) and the contralateral trunk lateral flexion (Healthy = 0.000°, Stroke = 4.50°) were only observed in stroke participants but not in healthy controls.
  When playing the shoulder horizontal abduction game, the stroke participant’s maximum compensation movements were significantly larger than the healthy control’s maximum deviation in ipsilateral shoulder hiking (z = − 2.45, p = 0.014, d = 0.51), ipsilateral shoulder depression (z = − 2.32, p = 0.022, d = 0.48), contralateral shoulder depression (z = 2.79, p = 0.006, d = 0.58), and trunk extension (z = − 3.82, p = 0.014, d = 0.80). It should be noted that the contralateral shoulder depression (Healthy = 0.000 m, Stroke = 0.015 m) and trunk extension (Healthy = 0.000°, Stroke = 4.50°) were only observed in stroke participants but not in healthy controls.
2. Differences between maximum deviations in healthy participants and the minimum compensation in stroke participants
  Table 2 lists the distance and angle differences between the minimum compensations in stroke participants and the maximum deviations in healthy controls. When playing the shoulder flexion game, the minimum compensation movements of stroke participants were significantly larger than the maximum deviation of healthy controls in the contralateral shoulder depression (z = − 3.49, p = 0.002, d = 0.73) and contralateral trunk lateral flexion (z = − 1.31, p = 0.044, d = 0.27). There were no significant differences found in other movements.
  When playing the shoulder horizontal abduction game, the minimum compensation movements of stroke participants were significantly larger than the maximum deviation of healthy controls in the contralateral shoulder depression (z = − 1.66, p = 0.031, d = 0.35), forward trunk flexion (z = − 2.48, p = 0.012, d = 0.52), trunk extension (z = − 3.67, p = 0.019, d = 0.77) and ipsilateral elbow flexion (z = − 2.54, p = 0.008, d = 0.53). There were no significant differences found in other movements.
3. Compensatory movements
  There were two steps to quantify the thresholds of compensatory movements. It was the first to identify any significant differences between the maximum deviation of healthy controls and the minimum compensation of stroke participants. If there were significant differences, the median of the minimum compensation of stroke participants was selected as the threshold to determine the compensation. Otherwise, the median of the maximum deviation of healthy controls would be the recommended threshold to determine the compensatory movement of stroke participants.

**Table 1:** Summary of maximum deviation of healthy controls and the maximum compensation of stroke participants in shoulder flexion and horizontal abduction

Movements	Shoulder flexion (Game 1)				p value	Shoulder horizontal abduction (game 2)				p value
	Maximum deviation in healthy participants	Maximum compensation in stroke participants	Mann- Whitney U	z value		Maximum deviation in healthy participants	Maximum compensation in stroke participants	Mann- Whitney U t	z value	
Ipsilateral shoulder hiking	3.00 cm (2.00–7.00 cm)	5.00 cm (3.70–13.00 cm)	32.0	− 2.27	0.025*	2.00 cm (2.00–5.00 cm)	4.00 cm (3.25–11.00 cm)	28.5	− 2.45	.014*
Contralateral shoulder hiking	1.00 cm (0.00–3.00 cm)	2.00 cm (0.00–3.00 cm)	49.0	− 1.49	0.173	1.00 cm (1.00–4.00 cm)	2.00 cm (1.25–5.00 cm)	55.5	− 1.12	.285
Ipsilateral shoulder depression	2.00 cm (0.00–4.00 cm)	4.50 cm (2.00–8.00 cm)	20.5	− 2.89	0.003*	1.00 cm (0.02–4.00 cm)	2.50 cm (2.00–5.00 cm)	31.0	− 2.32	.022*
Contralateral shoulder depression	0.00 cm (0.00–2.00 cm)	1.50 cm (1.00–7.00 cm)	9.5	− 3.93	0.000*	0.00 cm (0.00–5.00 cm)	2.50 cm (1.25–5.00 cm)	23.5	− 2.79	.006*
Ipsilateral trunk lateral flexion	0.00° (0.00–4.00°)	0.00° (0.00–5.00°)	78.0	0.00	1.00	0.00° (0.00–5.00°)	1.00° (0.00–8.00°)	65.0	− 0.71	.556
Contralateral trunk lateral flexion	0.00° (0.00–5.00°)	4.50° (2.25–8.00°)	25.5	− 2.80	0.008*	3.00° (1.25–8.00°)	6.50° (2.75–13.00°)	37.5	− 1.78	.078
Forward trunk flexion	2.50° (0.00–20.00°)	0.00° (0.00–21.00°)	75.0	− 0.16	0.906	6.00° (4.50–14.00°)	2.50° (0.00–19.00°)	56.0	− 1.07	.308
Trunk extension	0.50° (0.00–7.00°)	2.00° (0.75–10.00°)	53.0	− 1.27	0.244	0.00° (0.00–4.00°)	4.50° (0.75–8.00°)	28.0	− 3.82	.014*
Ipsilateral elbow flexion	5.50° (0.00–37.00°)	27.50° (17.25–57.00°)	32.5	− 2.21	0.025*	8.50° (4.50–49.00°)	4.50° (0.50–53.00°)	52.5	− 1.24	.225

Values are median (IQR: Q1–Q4)

*p < 0.05

**Table 2:** Summary of maximum deviation of healthy controls and the minimum compensation of stroke participants in Shoulder Flexion and Horizontal Abduction

Movements	Shoulder flexion (game 1)					Shoulder Horizontal Abduction (Game 2)				
	Maximum deviation in healthy participants	Minimum compensation in stroke participants	Mann- Whitney *U*	z value	p value	Maximum deviation in healthy participants	Minimum compensation in stroke participants	Mann- Whitney *U*	Z value	p value
Ipsilateral shoulder hiking	3.00 cm (2.00–7.00 cm)	1.50 cm (1.00–8.00 cm)	42.5	− 1.76	0.087	2.00 cm (2.00–5.00 cm)	1.00 cm (1.00–3.00 cm)	46.0	− 1.60	0.131
Contralateral shoulder hiking	1.00 cm (0.00–3.00 cm)	1.00 cm (0.25–2.00 cm)	66.0	− 0.62	0.588	1.00 cm (1.00–4.00 cm)	1.00 cm (1.00–2.00 cm)	61.0	− 0.86	0.436
Ipsilateral shoulder depression	2.00 cm (0.00–4.00 cm)	1.00 cm (1.00–2.00 cm)	69.5	− 0.43	0.689	1.00 cm (0.02–4.0 cm)	1.00 cm (1.00–3.00 cm)	68.0	− 0.50	0.655
Contralateral shoulder depression	0.00 cm (0.00–2.00 cm)	1.00 cm (1.00–1.00 cm)	18.0	− 3.49	0.002*	0.00 cm (0.00–5.00 cm)	1.00 cm (1.00–2.00 cm)	46.0	− 1.66	0.031*
Ipsilateral trunk lateral flexion	0.00° (0.00–4.00°)	0.00° (0.25–3.00°)	69.0	− 0.49	0.689	0.00° (0.00–5.00°)	0.00° (0.00–2.00°)	73.0	− 0.280	0.832
Contralateral trunk lateral flexion	0.00° (0.00–5.00°)	1.00° (1.00–2.00°)	53.5	− 1.31	0.044*	3.00° (1.25–8.00°)	1.50° (1.00–4.00°**)**	64.0	− 0.67	0.524
Forward trunk flexion	2.50° (0.00–20.00°)	0.00° (0.00–10.00°)	75.5	− 0.34	0.760	6.00° (4.50–14.00°)	0.50° (0.00–17.00°)	27.0	− 2.48	0.012*
Trunk extension	0.50° (0.00–7.00°)	2.00° (1.00–4.00°)	56.0	− 1.12	0.308	0.00° (0.00–4.00°)	1.00° (0.25–7.00°)	30.0	− 3.67	0.019*
Ipsilateral elbow flexion	5.50° (0.00–37.00°)	5.00° (4.00–15.00°)	69.5	− 0.41	0.689	8.50° (4.50–49.00°)	1.50° (1.00–13.00°)	25.5	− 2.54	0.008*

Values are mean (IQR: Q1–Q4)

*p < 0.05

Table 3 summarizes compensatory movements and thresholds found in both shoulder flexion and shoulder horizontal abduction. The three shoulder movements, including the ipsilateral shoulder hiking, the ipsilateral shoulder depression, and the contralateral shoulder depression, were observed in two planar motor exercises. A significant difference was found in contralateral shoulder depression when comparing the maximum deviation of the healthy controls with both the maximum and minimum compensations of stroke participants. The trunk compensatory movements were different in the two planar motor exercises. The contralateral trunk lateral flexion was observed in the shoulder flexion, while the trunk extension was observed in the shoulder horizontal abduction. The left column of Table 3 lists the joints matched in Kinect. The orientation and distance values of these matched joints could be used to calculate the thresholds of compensatory movements. These calculated thresholds would be less accurate than the recommended thresholds in Table 3 because of the low accuracy of Kinect. The divisions of two thresholds (calculated and recommended thresholds) could determine the constants mentioned in Step 2 of the compensation detector.

**Table 3:** Summary of compensatory movements

Joint		Shoulder Flexion		Shoulder Horizontal Abduction	
Kinect	VMC	Compensatory movements	Threshold	Compensatory movements	Threshold
Spine & Chest	Trunk	Contralateral trunk lateral flexion^a^	1.00°	Trunk extension^a^	1.00°
Chest & Shoulders (left and right)	Shoulder	Ipsilateral shoulder hiking	3.00 cm	Ipsilateral shoulder hiking	2.00 cm
		Ipsilateral shoulder depression	2.00 cm	Ipsilateral shoulder depression	1.00 cm
		Contralateral shoulder depression^a^	1.00 cm	Contralateral shoulder depression^a^	1.00 cm
Elbow	Elbow	Ipsilateral elbow flexion	5.50°		

^a^The movements observed in two comparisons

The results also showed that, during the five-minute VR game session, stroke participants had more distal compensatory movement patterns (CMPs), such as the ipsilateral shoulder hiking, during the first three minutes of gameplay, and after that, they had more proximal CMPs, such as the trunk extension. Moreover, the sums of compensatory movements’ numbers were different for each joint {trunk [mean = 49, (35–60)], shoulder [mean = 24, (22–35)], and elbow [mean = 6, (5–11)]}.

### Summary and discussion of study 1

The results of Study 1 found that stroke participants adopt various compensatory movements in two planar motor exercises. The results further quantified the thresholds to determine compensatory movements and revealed the pattern of compensatory movements. Both the findings and the quantitative results could be applied to improve the design of VRS.

## Compensatory movements in two planar motor exercises

Results showed that the trunk and shoulder compensatory movements were observed in stroke participants when they took two planar motor exercises. Compensatory movements in the shoulder flexion exercise were similar to those observed in the reaching task. For example, Fuller et al. [30] found that the stroke subjects leaned toward their non-reaching side to reduce the load on the shoulder elevator region, where the most profound effects of fatigue, such as muscle saturation, were observed. Furthermore, Cirstea and Levin [10] pointed out that the weakness of the shoulder muscle could increase trunk compensation.

The compensatory movement of the elbow was only observed in the shoulder flexion but not in the shoulder horizontal abduction. It could be because the primary agonist muscles for horizontal abduction are around the shoulder but not the elbow. Thus, the elbow muscles were not saturated in the repetitive task of shoulder horizontal abduction, even though the elbow muscle was weak in stroke participants.

## Patterns of compensatory movements

During the five-minute gameplay in this study, the stroke participants firstly showed more distal CMPs and later more proximal CMPs after around three minutes. The change of CMPs could be due to the repetitive motion-induced shoulder muscle fatigue, reflecting the muscle weakness in stroke participants. The time of shoulder fatigue was similar to that found in a previous study's repetitive shoulder reaching task [30].

The CMPs found in this study during the shoulder flexion exercise are similar to the CMPs found in the traditional shoulder reaching task. McCrea et al. [13] identified that stroke individuals tend to use elbow flexion during early movements in reaching (shoulder flexion with arm flexed to 90^0^) as it reduces the lever distance between the shoulder and the center of arm mass and further reduces the need to generate the higher power to execute the movement.

The results showed that compensatory movements were still present after stroke individuals had taken rehabilitation for at least three months. The study [31] found that the compensatory reaching involving the trunk could be maintainable even for one year. It also suggests that training involving a conscious decision to control the trunk compensation may lead to ideal restorative strategies. Thus, the developed compensation-aware VRS should detect compensatory movements and promote stroke participants' awareness of controlling compensatory movements.

## Implications for improving the design of VRS

The results of Study 1 could be applied to the design of VRS in twofold. First, the quantitative thresholds of compensatory movements recommended in Table 3 would be deployed in Step 2 of the compensation detector of VRS. However, the thresholds might need to be modified to compensate for the low accuracy of Kinect [16, 32]. Hence, some constants (e.g., β) proposed in Step 2 multiply the calculated distances of orientation or distance in Kinect to be compared with the recommended thresholds.

Second, the duration of the exercise game needs to be shortened to less than three minutes, which could reduce the shoulder muscle fatigue and the proximal CMPs. Then the VRS could focus on the distal CMPs to improve the quality of UE movement.

## Study 2: preliminary validation of the VRS

The purpose of Study 2 was to provide preliminary validation for the VRS deployed in a community rehabilitation center, where the design of VRS has been improved based on the findings in Study 1. The validation focuses on the VRS's capacity to improve UE motor functions and detect compensatory movements. Two research hypotheses would be tested in this study. First, the UE motor functions in older adults with stroke would be significantly improved after the VRS-based training. Thus, the participants' UE motor functions would be measured pre- and post-intervention and then compared. Moreover, the UE motor functions after the VRS-based training would be compared with those after the conventional training. The comparison could not only testify to the first research hypothesis but also explore the benefit of VRS technology brought to motor rehabilitation.

Second, the VRS could effectively detect compensatory movements in the community rehabilitation center, which could be proved by the number of compensatory movements collected in Study 2. Moreover, the number of compensatory movements would be collected pre- and post-intervention and then compared too. The comparison would provide insights on whether the compensation-aware VRS could assist older adults with stroke by reducing compensatory movements.

### Participants

Eighteen participants who satisfied the inclusion criteria and fulfilled the prerequisites in the screen test were recruited. Participants were randomly allocated to the VR group (n = 9) or the conventional training (CT) group (n = 9). Three subjects dropped out during the study, in which one from the CT group due to a recent diagnosis of dementia (n = 1) and two from the VR group due to uncontrolled hypertension (n = 1) and hemiplegic shoulder pain secondary to non-physiotherapy related intervention (n = 1), respectively. A total of fifteen subjects (8 control and 7 experimental) remained at the end of the study.

Inclusion criteria: (1) age 55 years old and above, (2) at least 6 months post-stroke secondary to ischemic/hemorrhagic stroke with chronic hemiparesis, (3) discharged from an acute hospital, medically stable hypertension and/or high cholesterol and/or diabetes, (4) no pre-existing shoulder joint conditions (e.g., osteoarthritis, fracture) of the hemiparetic upper-extremity, (5) no ataxia and cerebellar symptoms, (6) no systemic conditions such as rheumatoid arthritis, (7) no cognitive impairments or history of psychiatric condition.

Screen test: active range of motion (AROM) of shoulder flexion at least 45°, able to grip the stylus of the haptic device with the affected hand, able to follow instructions, no orthopedic changes or pain on the affected upper extremity, no sensation deficit, no proprioception deficit, no visual or auditory deficit, no increase in muscle tone.

### Experiment setup and procedure

The study was conducted at a local community rehabilitation center, as shown in Fig. 1. The setup of VRS was the same as that in Study 1. Each group underwent ten sessions of an additional 12 min of either conventional therapy or VR games on top of their usual rehabilitation schedule in three months. Measurements of Wolf Motor Function Test (WMFT), Fugl-Meyer Assessment—Upper Extremity (FMA-UE), range of motion (ROM), Stroke Rehabilitation Motivation Scale (SRMS), as well as the number of compensatory movements of the subjects in the VR group were collected through the VRS before and after the intervention for comparison. Three of the participants from the CT group were excluded from the measurement of the number of compensatory movements due to inability to sustain grip on the stylus (n = 2) and keratoconjunctivitis sicca (n = 1). Before the intervention, a familiarization trial on the VRS was conducted for the VR group.

Participants in the VR group underwent two 3-min VR games with a 6-min rest in between. To ensure safety, participants playing the VR games should notify the experimenters immediately if they experienced any symptoms of cybersickness or discomfort. In the CT group, each participant experienced an additional 3 min of their usual rehabilitation exercises, with a 6-min rest in between. To make sure participants were medically stable throughout each session, their blood pressure and heart rate were also routinely taken pre-and post-intervention.

### Data analysis

Data were analyzed in IBM SPSS Statistics Version 21. As the data analyzed was not normally distributed, median values and interquartile range of the scores obtained from the outcome measures were calculated. Mann Whitney *U* test was used to determine if there was a significant difference in the improvements in shoulder function and quality of movements between control and experimental groups. Wilcoxon matched paired test was used to compare any significant improvements in shoulder function and quality of movements between pre-intervention and post-intervention. The significance level was chosen at 0.05.

## Results

1. Participant's characteristics
  The median age of participants was 74 years (range 63 to 79.5 years), and the median time for stroke chronicity was two years (range 1.5 to 5.5 years). There were no significant differences (p > 0.05) in baseline measures of all outcome measures, and there were also no significant differences between the two groups with respect to gender, comorbidities, affected side, handedness, and stroke type, except for age (p = 0.047). The participant characteristics and baseline tests are summarized in Table 4.
2. UE motor functions: FMA, WMFT & ROM
  Table 5 summarizes the three major tests on UE motor functions. First, there was a statistically significant improvement in both VR (z = − 1.693, p = 0.045, d = 0.640) and CT (z = − 1.706, p = 0.044, d = 0.603) groups in the total FMA-UE score, but there was no significant difference between the two groups (z = − 0.763, p = 0.231, d = 0.197) post-intervention.
  Second, for WMFT, participants in the VR group demonstrated a statistically significant improvement in FAS (z = − 1.807, p = 0.009, d = 0.683) and TIME (z = − 2.366, p = 0.035, d = 0.836) as compared to the baseline. There were no significant differences between VR and CT groups in FAS and TIME after the intervention. It was surprising to note a statistically significant increase in WMFT- weights in the CT group (Z = − 1.511, p = 0.020, d = 0.534), but not in the VR group. But there was no statistically significant difference in the grip strength in both the VR and CT groups. Further trend analysis demonstrated no decline in weight-carrying performance and grip strength post the VR intervention.
  Lastly, both groups had no statistically significant improvements in the ROM of shoulder flexion, horizontal abduction, and horizontal adduction, elbow flexion. Although there were no remarkable improvements in the ROM, the results indicated the maintenance of the ROMs of both shoulder and elbow after the VR intervention.
3. SRMS
  There was a significant (z = − 1.334, p = 0.000, d = 0.344) increase of 28.6% in the VR group who agreed that participating in rehabilitation was exciting. The CT group paled in comparison with an increase of 25%. The percentage of subjects who disagreed with decreasing their motivation for rehabilitation increased from 71.4% to 100% in the VR group. In contrast, the percentage of subjects in the CT group who disagreed with the statement dropped from 62.5% to 50%.
  There was no significant improvement in the SRMS total score for the VR and CT groups. There was also no significant difference between the two groups. There was an observed increase of 3 points in the VR group's intrinsic motivation compared to the 1-point increase in the CT group.
4. Number of compensatory movements
  All compensatory movements listed in Table 3 were detected in the present study. Table 6 lists the numbers of ipsilateral elbow flexion observed in pre- and post- VR intervention. The reduction of the number of ipsilateral elbow flexion was promising. The table also lists the total number of compensatory movements (the sum of all compensatory movements detected by the VRS), where its medians were reduced by 14 in Game 1 and 22 in Game 2, respectively. However, the number of each compensatory movement was not significantly reduced after the VR intervention.

**Table 4:** Characteristics of participants and the baseline of function status before the experiment

Characteristics	VR group	CT group	Baseline function status	VR group	CT group	Between group p-value
Age in years/median	74 (63–79)	83.5 (72–85)	FMA-UE			
Stroke in years/median	2 (1–4)	2 (2–6)	Upper extremity	31 (26.5–34)	35.5 (30–36)	0.076
Gender			Wrist	9 (6–10)	10 (5–11)	0.116
Female, n(%)	3 (20)	2 (13.33)	Hand	13 (11–14)	13 (10–14)	0.306
Male, n(%)	4 (26.7)	6 (40)	Coordination	4 (3.5–6)	5(3.5–6)	0.306
Comorbidities			Total	59 (48–61)	64 (50–65)	0.076
Hypertension, n(%)	7 (46.67)	7 (46.67)	WMFT			
Diabetes Mellitus, n(%)	5 (33.33)	1 (6.67)	FAS	69 (63.5–71.5)	72.5 (57–74.5)	0.14
Dyslipidemia, n(%)	6 (40)	3 (20)	Time in seconds	3.24 (2.71–3.45)	2.78 (1.61–3.385)	0.23
Atrial fibrillation, n(%)	0	2 (13.33)	SRMS	26 (22–27.5)	23 (20.5–23.5)	0.076
Stroke type			ROM in degree			
Ischaemic, n(%)	6 (40)	7 (46.67)	Shoulder flexion	130 (110–132.5)	130 (110–132.5)	0.477
Haemorrhagic, n(%)	1 (6.67)	1 (6.67)	Shoulder horizontal Abduction	20 (10–23.5)	20 (10–23.5)	0.168
Affected side			Shoulder horizontal Adduction	95 (77–97)	95 (77–97)	0.116
Right, n(%)	0	1 (6.67)				
Left, n(%)	7 (46.67)	7 (46.67)				

Values in baseline are median (IQR, Q1-Q4)

**Table 5:** Summary of test results of FMA-UE, WMFT and ROM

Upper extremity outcome measure	VR Group			CT Group			Between Groups
	Median (IQR)		p-value	Median (IQR)		p-value	p-value
	Pre-test	Post-test		Pre-test	Post-test		
FMA-UE							
Upper extremity	31 (26.5–34)	33 (32–34.5)	0.065	35.5 (30–36)	35 (31–36)	0.142	0.168
Wrist	9 (6–9)	9 (8–9.5)	0.207	10 (5–10)	10 (8.5–10)	0.1585	0.500
Hand	14 (11–14)	14 (13–14)	0.051	13 (10–14)	14 (10.5–14)	0.168	0.477
Coordination	4 (3.5–6)	6 (3.5–6)	0.128	6 (3.5–6)	5.5 (3.5–6)	0.352	0.198
Total score	59 (48–61)	61 (58–63)	0.045*	64 (50–65)	64.5 (53.5–66)	0.044*	0.231
WMFT							
Functional ability score (FAS)	69 (63.5–71.5)	73 (69–73.5)	0.009*	72.5 (57–74.5)	74 (60–74.5)	0.080	0.076
Time in seconds	3.24 (2.71–3.45)	1.95 (1.47–2.65)	0.035*	2.78 (1.61–3.385)	1.75 (1.33–3.73)	0.065	0.198
Weights in lbs	7 (4–8.5)	7 (4–9.5)	0.500	8 (4–9)	8.5 (4.5–10)	0.020*	0.094
Grip strength in kg	10.3 (8.42–14.5)	12 (7.835–13.5)	0.376	15.5 (8.49–17.99)	16.33 (9.75–20.82)	0.181	0.198
ROM							
Shoulder flexion in degree	130 (110–132.5)	124(108–129.5)	0.263	124.5(108.5–133.5)	129.5(109–139.5)	0.363	0.306
Shoulder horizontal abduction in degree	20 (10–23.5)	24 (9–25.00)	0.116	21.5(13.5–32.5)	26 (20–27.5)	0.336	0.433
Shoulder horizontal adduction in degree	95(77–97)	104 (89.5–107)	0.172	100.5 (82–107)	107 (90.5–115)	0.198	0.191
Elbow flexion in degree	140 (134.5–143)	141 (135.5–144)	0.399	141 (130.5–142.5)	144.5 (140–145.5)	0.075	0.232

Values are median [IQR, Q1–Q4)]

*p < 0.05

**Table 6:** Numbers of compensatory movements observed in Pre- and Post- VR intervention

	VR group		
	Pre-intervention	Post-intervention	p-value
Shoulder flexion—game 1			
Number of ipsilateral elbow flexion	9 (3–34)	1 (0–11)	0.088
Total number of compensatory movements	81 (54–110)	67 (22–83)	0.231
Shoulder horizontal abduction—game 2			
Total number of compensatory movements	67 (46.67)	45 (22–74)	0.337

## Discussion

The results of Study 2 indicate that the VRS-based training could significantly improve the UE motor function of participants, which supported the first research hypothesis of Study 2. Furthermore, the results also suggested that the VRS-based training could achieve similar outcomes of UE rehabilitation as the conventional training but had an advantage in engaging participants in motor training. The results also demonstrate that the developed VRS could detect compensatory movements in stroke participants. However, the number of compensatory movements was not significantly reduced after the VRS-based training.

1. VRS and the recovery of UE motor functions
  Table 5 shows that the training through the present VRS could significantly improve the participant's motor functions, as measured in FMA-UE (total score) and WMFT (FAS and time). Thus, the present VRS was proved to support older adults with stroke to improve UE motor functions, which supported the first research hypothesis of this study. Furthermore, the training through the present VRS could be comparable with the conventional training in enhancing the recovery of UE motor impairments since there were no significant differences in FMA-UE, WMFT, and ROM measurements between the VR and CT groups. Levin et al.[33] suggested that the VR training could help achieve motor relearning via adaptive neuroplasticity changes, leading to gains in motor function.
  However, the results of Study 2 could not provide evidence that the present compensation-aware VRS was better than the previous VRS in recovering UE motor functions. Although the previous VRS could not detect the compensation, they could achieve a similar level of motor function recovery as the conventional training [3]. In other words, the compensation detection and feedback might not bring additional advantages to the VRS to enhance the recovery of UE motor functions in the short term (such as three months). Likely, the increases in the training duration and repetition during VR sessions contribute to the improved motor ability of the hemiparetic UE [34]. The future study needs to explore how the compensation-aware VRS improves the quality of UE movements in the long term.
2. Engagement in VR training
  An observable trend demonstrated more improvements in intrinsic motivation scores in the VR group than in the CT group, as shown by the SRMS measurements. Our results were consistent with the previous research findings [35], where the highest intrinsic motivation was found in subjects of the VR group. Despite traditional beliefs that the elderly possess negative attitudes towards technology [36], the introduction of VR gaming could enhance engagement and motivation. Besides the engaging visual and audio contents in VR scenes, the present VRS incorporates a goal-oriented reward scheme to motivate older adults with stroke, such as collecting coins to gain game scores. The reward scheme could positively impact stroke participants' motivations to complete the game task, which might increase their intention to be aware of compensatory movements.
3. Validation of the compensation detector
  The number of compensatory movements indicates that the VRS could detect the compensatory movements, which supported the second research hypothesis of this study. The results proved that the designed compensation detector could successfully detect compensatory movements via online processing of the data stream from the Kinect. The results also suggested the feasibility of deploying the compensation-aware VRS in a community rehabilitation center.
  However, the reduction of compensatory movements was not significant in this study, as shown in Table 6, which could be partially attributed to the design of some feedback. For example, the punishment of game scores was unexpected to motivate a few participants to ignore the visual feedback on compensatory movements in their haste to obtain high game scores. Such ignorance could lead to a lack of self-correction in compensatory movements. Therefore, future research needs to improve the design of the audio-visual feedback to guide stroke individuals to reduce compensatory movements.
4. VRS and telerehabilitation
  The development of such VRS is essential in the current social context, given the high prevalence of chronic stroke patients with persistent UE impairments [4], and only 5% of them fully recover the hemiparetic upper extremity [37]. Moreover, there is a short supply of workforce resources and limited therapist-patient interaction time [6] in the community to meet the increasing global demand of a rising aging population. Since the developed VRS could be used as one telerehabilitation form in the community, older adults with stroke could take motor rehabilitation in a comfortable and familiar environment [38].

## Limitations

Some limitations are present in the current study and need to be acknowledged. The main limitation is the small sample size. The ideal sample size was 136 (calculated in G*Power Version 3.1.9.2) in Study 2 aimed to improve upper limb motor function in stroke rehabilitation through VRS, as required in the previous research [3]. In addition, the study was implemented in a single community rehabilitation center. The community rehabilitation center did not have enough eligible participants since the center only serves the older adults in one community. Moreover, not all older adults with stroke in this center could satisfy the inclusion criteria and fulfilled the prerequisites in the screen test. The short staffing of this center could also not support conducting a long-term experiment to recruit more participants. Hence the results may not be a good representation of the general stroke population in Singapore.

The second limitation of this study is the missing resistance training exercise of VRS. Although the haptic device used in the study can provide kinesthetics feedback to users in the form of vibration and force feedback, it is not comparable to the equipment for strength training in conventional therapy. Nonetheless, it is encouraging to find out that there was no compromise in the upper-extremity muscle strength and the grip strength in VR participants after the study.

The third limitation of this study is the lack of formal assessment of cognitive functions when recruiting the participants. The assessment of cognitive impairment was based on participants' medical records collected in the community rehabilitation center, which could not provide valid evidence for assessing participants' cognitive impairment.

## Conclusion

The present study developed a compensation-aware VRS for UE rehabilitation in the community. The VRS can detect compensatory movements of the trunk, shoulder, and elbow, and the VRS-based training could be comparable with conventional training in enhancing the recovery of UE motor functions. Areas requiring improvements are identified, and future studies should establish the long-term intervention with more sample participants to determine the efficacy of the developed VRS.

## Supplementary Information

**Additional file 1:** Player’s performances on playing Shoulder Flexion Game.

## Data Availability

The datasets analyzed during the current study are available from the corresponding author on reasonable request.

## References

[1] Langhorne P, Bernhardt J, Kwakkel G (2011). Stroke rehabilitation. Lancet.

[2] Coupar F, Pollock A, Rowe P, Weir C, Langhorne P (2012). Predictors of upper limb recovery after stoke: a systematic review and meta-analysis. Clin Rehabil.

[3] Turolla A, Dam M, Ventura L, Tonin P, Agostini M, Zucconi C (2013). Virtual reality for the rehabilitation of the upper limb motor function after stroke: a prospective controlled trial. J NeuroEng Rehabilitation.

[4] Jones L, van Wijck F, Grealy M, Rowe P, Jones L (2011). A changing stroke rehabilitation environment: implications for upper limb interventions. 5th International conference on pervasive computing technologies for healthcare (pervasivehealth) and workshops 2011.

[5] McCue M, Fairman A, Pramuka A (2010). Enhancing quality of life through telerehabilitation. Phys Med Rehabilitation Clin N Am..

[6] Bower K, Gustafsson L, Hoffmann T, Barker R (2012). Self-management of upper limb recovery after stroke: how effectively do occupational therapists and physiotherapists train clients and careers?. Br J Occup Ther.

[7] Threapleton K, Drummond A, Standen P (2016). Virtual rehabilitation: what are the practical barriers for home-based research?. Digit Health.

[8] Merdler T, Liebermann DG, Levin MF, Berman S (2013). Arm-plane representation of shoulder compensation during pointing movements in individuals with stroke. J Electromyogr Kinesiol.

[9] Liu W, Waller SM, Kepple T, Whitall J (2013). Compensatory arm reaching strategies after stroke: induced position analysis. J Rehabil Res Dev.

[10] Cirstea MC, Levin MF (2000). Compensatory strategies for reaching in stroke. Brain.

[11] Lang C, Bailey R, Schaefer S, Birkenmeier R (2012). Assessment of upper limb impairment, function, and activity after stroke: foundations for clinical decision making. J Hand Ther.

[12] van Kordelaar J, van Wegen E, Nijland R, Daffertshofer A, Kwakkel G (2013). Understanding adaptive motor control of the paretic upper limb early poststroke: the EXPLICIT-stroke program. Neurorehabil Neural Repair.

[13] McCrea P, Eng J, Hodgson A (2005). Saturated muscle activation contributes to compensatory reaching strategies following stroke. J Neurophysiol.

[14] Roby-Brami A, Feydy A, Combeaud M, Biryukova E, Bussel B, Levin M (2003). Motor compensation and recovery for reaching in stroke individuals. Acta Neurol Scand.

[15] Levin MF, Kleim J, Wolf SL (2009). What do motor "recovery" and "compensation" mean in individuals following stroke?. Neurorehabil Neural Repair.

[16] Reither FM, Migotsky LR, Haddix N, Engsberg C (2017). Upper limb movement reliability and validity of the kinect version 2. Disabil Rehabil Assist Technol.

[17] Foreman MH, Engsberg JR (2019). A virtual reality tool for measuring and shaping trunk compensation for persons with stroke: design and initial feasibility testing. J Rehabil Assist Technol Eng.

[18] Valdés BA, Glegg SMN, Van der Loos HFM (2017). Trunk compensation during bimanual reaching at different heights by healthy and hemiparetic adults. J Mot Behav.

[19] Cai S, Li G, Zhang X, Huang S, Zheng H, Ma K (2019). Detecting compensatory movements of stroke survivors using pressure distribution data and machine learning algorithms. J Neuroeng Rehabil.

[20] Cai S, Wei X, Su E, Wu W, Zheng H, Xie L (2020). Online compensation detecting for real-time reduction of compensatory motions during reaching: a pilot study with stroke survivors. J Neuroeng Rehabil.

[21] Joost VK, Van Wegen EEH, Gert K (2012). Unraveling the interaction between pathological upper limb synergies and compensatory trunk movements during reach-to-grasp after stroke: a cross-sectional study. Exp Brain Res.

[22] Combs S, Finley M, Henss M, Himmler S, Lapota K, Stillwell D (2012). Effects of a repetitive gaming intervention on upper limb impairments and function in persons with chronic stroke: a preliminary study. Disabil Rehabil.

[23] Park M, Ko MH, Oh SW, Lee JY, Ham Y, Yi H, Choi Y, Ha D, Shin JH (2019). Effects of virtual reality-based planar motion exercises on upper extremity function, range of motion, and health related quality of life: a multicenter, single blinded, randomized, controlled pilot study. J NeuroEng Rehabilitation..

[24] Clark RA, Pua YH, Fortin K, Ritchie C, Webster KE, Denehy L (2012). Validity of the microsoft kinect for assessment of postural control. Gait Posture.

[25] Alankus G, Kelleher C (2015). Reducing compensatory motions in motion-based video games for stroke rehabilitation. Hum Comp Interact.

[26] Payton C, Bartlett R (2008). Biomechanical evaluation of movement in sport and exercise.

[27] Jaspers E, Feys H, Bruyninckx H, Harlaar J, Molenaers G, Desloovere K (2011). Upper limb kinematics: development and reliability of a clinical protocol for children. Gait Posture.

[28] Siliconcoach, Siliconcoach Pro 7.022 start guide. 2010.

[29] Cronin J, Nash M, Whatman C (2006). Assessing dynamic knee joint range of motion using siliconcoach. Phys Ther Sport.

[30] Fuller JR, Lomond KV, Fung J, Côté JN (2009). Posture-movement changes following repetitive motion-induced shoulder muscle fatigue. J Electromyogr Kinesiol.

[31] Thielman G (2013). Insights into upper limb kinematics and trunk control one year after task-related training in chronic post-stroke individuals. J Hand Ther.

[32] Mousavi HH, Khademi M (2014). A review on technical and clinical impact of microsoft kinect on physical therapy and rehabilitation. J Med Eng.

[33] Levin MF, Weiss P, Keshner EA (2015). Emergence of virtual reality as a tool for upper limb rehabilitation: Incorporation of motor control and motor learning principles. Phys Ther.

[34] Sucar LE, Orihuela-Espina F, Velazquez RL, Reinkensmeyer DJ, Leder R, Hernández-Franco J (2014). Gesture therapy: an upper limb virtual reality-based motor rehabilitation platform. IEEE Trans Neural Syst Rehabil Eng.

[35] Fan S, Su F, Chen S, Hou W, Sun J, Chen K, Lin W, Hsu S (2014). Improved intrinsic motivation and muscle activation patterns in reaching task using virtual reality training for stroke rehabilitation: a pilot randomized control trial. J Med Biol Eng.

[36] Iyer R, Eastman JK (2006). The elderly and their attitudes toward the Internet: the impact on Internet use, purchase, and comparison shopping. J Market Theory Pract.

[37] Tsu AP, Abrams GM, Byl NN (2014). Post stroke upper limb recovery. Semin Neurol.

[38] Lockery D, Peters JF, Ramanna S, Shay BL, Szturm T (2011). Store-and-feed forward adaptive gaming system for hand-finger motion tracking in telerehabilitation. Inf Technol Biomed.

